# CRISPR/Cas9-based depletion of 16S ribosomal RNA improves library complexity of single-cell RNA-sequencing in planarians

**DOI:** 10.1186/s12864-023-09724-4

**Published:** 2023-10-20

**Authors:** Kuang-Tse Wang, Carolyn E. Adler

**Affiliations:** Department of Molecular Medicine, Cornell University College of Veterinary Medicine, Ithaca, NY USA

**Keywords:** scRNA-seq, Ribodepletion, CRISPR/Cas9, DASH, Planarians

## Abstract

**Background:**

Single-cell RNA-sequencing (scRNA-seq) relies on PCR amplification to retrieve information from vanishingly small amounts of starting material. To selectively enrich mRNA from abundant non-polyadenylated transcripts, poly(A) selection is a key step during library preparation. However, some transcripts, such as mitochondrial genes, can escape this elimination and overwhelm libraries. Often, these transcripts are removed in silico, but whether physical depletion improves detection of rare transcripts in single cells is unclear.

**Results:**

We find that a single 16S ribosomal RNA is widely enriched in planarian scRNA-seq datasets, independent of the library preparation method. To deplete this transcript from scRNA-seq libraries, we design 30 single-guide RNAs spanning its length. To evaluate the effects of depletion, we perform a side-by-side comparison of the effects of eliminating the 16S transcript and find a substantial increase in the number of genes detected per cell, coupled with virtually complete loss of the 16S RNA. Moreover, we systematically determine that library complexity increases with a limited number of PCR cycles following CRISPR treatment. When compared to in silico depletion of 16S, physically removing it reduces dropout rates, retrieves more clusters, and reveals more differentially expressed genes.

**Conclusions:**

Our results show that abundant transcripts reduce the retrieval of informative transcripts in scRNA-seq and distort the analysis. Physical removal of these contaminants enables the detection of rare transcripts at lower sequencing depth, and also outperforms in silico depletion. Importantly, this method can be easily customized to deplete any abundant transcript from scRNA-seq libraries.

**Supplementary Information:**

The online version contains supplementary material available at 10.1186/s12864-023-09724-4.

## Background

Recent advances in single-cell transcriptomics (scRNA-seq) have greatly facilitated the exploration of cell type complexity. By capturing and barcoding mRNA on a cell-by-cell basis, scRNA-seq enables the identification of different cell types in a wide range of species. However, an important aspect of scRNA-seq is quality control in the analysis, which involves removing potential confounding factors such as sequencing depth and the percentage of mitochondrial reads [1, 2]. While these factors can be normalized in silico, it is optimal to minimize unwanted transcripts during library preparation to improve efficiency and more accurately probe cell complexity.

To maximize retrieval of protein-coding transcripts, most scRNA-seq methods capture mRNA by poly(A) enrichment [3, 4]. Ideally, poly(T) primers selectively bind to polyadenylated protein-coding transcripts to enable reverse transcription. This approach effectively eliminates ribosomal RNA, which comprises > 80% of total RNA. Despite its utility, poly(A) enrichment also prevents the capture of some informative RNA species, including histone mRNA, miRNA, and enhancer RNAs [5]. In addition, unwanted transcripts such as mitochondrial RNA leak out of damaged cells and are carried through into cDNA libraries [2]. These contaminants often appear as ambient RNA, a computational challenge for cell calling algorithms that can introduce bias into normalization [6, 7]. Due to these issues, poly(A) enrichment can distort the global transcriptional landscape.

Alternative ribodepletion methods have recently been developed for single-cell transcriptomics. For example, VASA-seq generates cDNA by hexamer primers with a T7 handle to enable subsequent RNA amplification by in vitro transcription. Ribosomal RNAs will then be removed from amplified RNA by rDNA probes and RNAse-H-mediated digestion [8]. RamDA-seq uses specific hexamers that are not present in rRNA to exclude it from downstream steps [9, 10]. However, these methods require new chemistry and reagents, raising the entry barrier for in-house setup.

Depletion of Abundant Sequences by Hybridization (DASH) is a CRISPR/Cas9-based method to remove unwanted DNA sequences from any DNA library [11]. With the customized design of single guide RNA (sgRNA), Cas9 can precisely remove unwanted cDNA sequences, and further enhance the detection of rare transcripts. DASH has not only been shown to efficiently deplete unwanted sequences in 16S sequencing and bulk RNA-seq [12, 13] but has been adapted to single-cell transcriptome methods, including scCLEAN, Smart-seq-total and MATQ-seq [14–17]. However, whether this depletion impacts library complexity or other metrics of sequencing quality has yet to be tested systematically.

Planarian mitochondrial 16S rRNA sequences are known to make up ~ 30% of bulk RNA-seq libraries generated by poly(A) enrichment [18]. In scRNA-seq experiments, most studies remove 16S from the analysis, but to what extent 16S rRNA contaminates scRNA-seq remains elusive [19, 20]. Here, we reanalyzed published datasets and found that unique molecular identifiers (UMIs) mapping to 16S constitute approximately 5–74% of sequencing reads regardless of the single-cell library generation strategy. We adapted DASH, which leverages CRISPR to remove unwanted sequences, to the 10X Chromium protocol to deplete mitochondrial 16S cDNA from planarian sequencing libraries [18]. We sequenced the same library with and without DASH treatment and carried out a side-by-side analysis to determine the impact of DASH treatment on overall scRNA-seq performance. We show that our protocol specifically depletes more than 90% of 16S UMIs from both cells and ambient RNA. This depletion increases the number of genes and non-16S UMIs detected per cell which improves the downstream analysis. We conclude that DASH can enhance library complexity, boosting the information retrieved from scRNA-seq experiments, with significant economic benefits. Importantly, this technique can be easily customized to deplete any abundant transcript after single-cell libraries are generated.

## Results

### Mitochondrial 16S transcript dominates reads in planarian scRNA-seq datasets

**Fig. 1:**
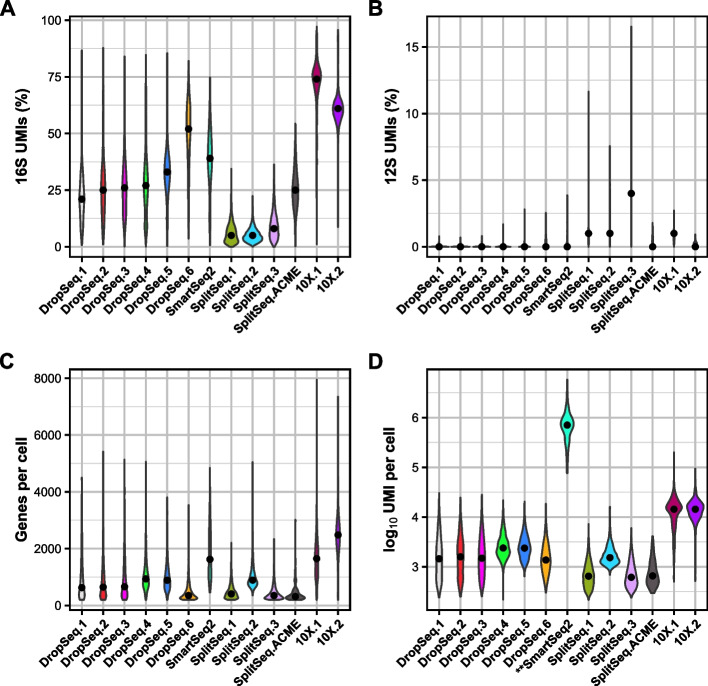
Planarian 16S rRNA is highly enriched in various scRNA-seq platforms. **A**, **B** Violin plots show the percentage of 16S UMIs (**A**) and 12S UMIs (**B**) across different published scRNA-seq datasets. **C**, **D** Violin plots show the number of genes (**C**) and UMIs (**D**) per cell. Note, SmartSeq2 does not implement UMI. The values graphed indicate total read counts for SmartSeq2. DropSeq.1–5: “head” datasets [19]; DropSeq.6:gfp(RNAi) [21]; SmartSeq2 [22]; SplitSeq.1–3: 0 days post-amputation (dpa), 1dpa and 2dpa [23]; SplitSeq.ACME [24]; 10X.1: post-pharyngeal wound fragments, 0 h [25]; 10X.2: X1 cells[20]

Planarians were one of the first animal models to be profiled at the whole organismal level using scRNA-seq. Because several different sequencing strategies have been applied to planarians, we first asked which method has the highest library complexity and the least 16S rRNA contamination. Thus, we collected 13 datasets from 7 studies using Drop-seq [19, 21], Smart-seq2 [22], Split-Seq [23], Split-Seq with ACME fixation [24], and 10X Chromium [20, 25] (Fig. 1). All methods use poly(T) primers to capture transcripts except for Split-Seq, which uses a mixture of random hexamers and poly(T) primers. To standardize these comparisons across libraries, we used the same pipeline to pre-process the barcodes and UMI tagging, align the reads to the genome (including the mitogenome), and generate quality metrics using the same parameters [26, 27].

To assess the abundance and quality of these different sequencing strategies, we applied several metrics. First, we analyzed the percentage of UMIs that mapped to the 16S transcript. Libraries made with 10X Chromium had the highest percentage of UMIs mapping to this transcript (61% and 74% on average) (Fig. 1A). By contrast, SplitSeq libraries were the lowest (ranging from 5 to 8%). DropSeq and ACME-based strategies ranged from 21 to 52%. The prevalence of 16S was unique because another mitochondrial rRNA, 12S, was consistently low across all datasets (Fig. 1B). These findings showed that in various scRNA-seq methods, the single 16S transcript accounted for a significant fraction of UMIs. Although Split-Seq appears to have the lowest percentage of UMIs mapping to the 16S, these libraries also had the highest number of unmappable reads (Supplementary Table 1). Next, we examined library complexity by assessing the number of genes and UMIs per cell. While 10X Chromium retained the highest levels of 16S UMIs as compared to other tested library methodologies, it also yielded the greatest library complexity, as assessed by the genes and UMIs detected per cell (Fig. 1C, D). In conclusion, these results show that 16S contamination is a severe problem in scRNA-seq preparation for planarians.

Because all of the scRNAseq library strategies rely on poly(A) enrichment, we hypothesized that 16S may be retained because of either polyadenylation or an internal polyA stretch [28, 29]. To distinguish between these two possibilities, we analyzed the coverage of reads across the 16S locus in one of our datasets using 10X Chromium. We found that the reads from a 10X dataset were strongly skewed to the 3’ end (Supplementary Fig. 1A), similar to other protein-coding genes, suggesting polyadenylation of 16S (Supplementary Fig. 1B). We also identified poly(A) stretches close to the middle of the 16S sequence (Supplementary Fig. 1B). Although these data did not provide a clear reason for the high abundance of 16S rRNA, we sought an alternative method to exclude 16S rRNA for scRNA-seq preparation.

### DASH effectively depletes 16S cDNA in scRNA-seq libraries

**Fig. 2:**
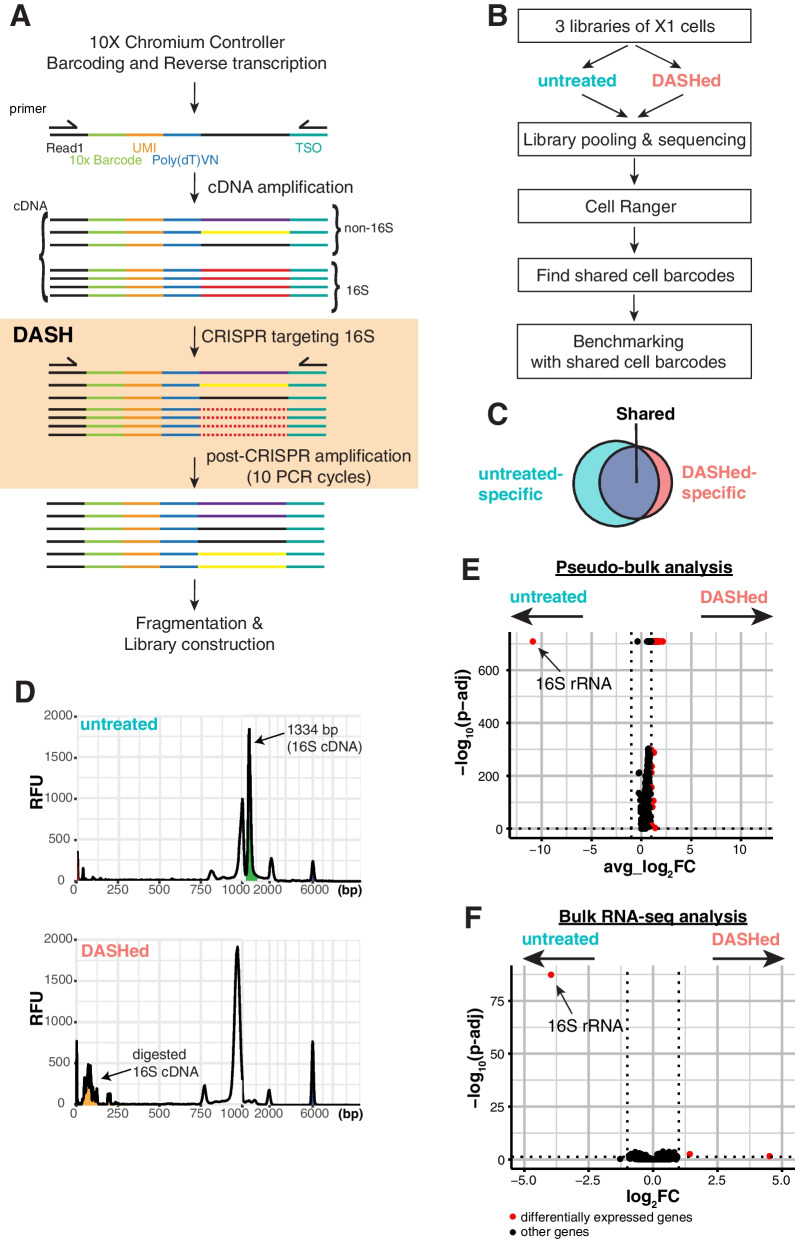
Workflow and depletion of 16S sequence with DASH. **A** Schematic of DASH protocol. The orange box highlights the DASH-specific steps, and all other steps are in the standard 10X protocol. Probes containing poly(T)VN, where V is any base except T, and N is any base, are used for poly(A) enrichment. cDNA is reverse-transcribed in the 10X Chromium Controller and amplified by cDNA primers, Read1 and template switching oligo (TSO). After generation of cDNA, 16S is depleted by incubation with Cas9 and sgRNAs targeting the 16S sequence, followed by post-CRISPR amplification with the same cDNA primers. The re-amplified cDNA is then fragmented and indexed for subsequent sequencing. **B** Schematic of experimental design to benchmark the performance of DASH. Three biological replicates of stem cells (X1 cells) are sorted and processed for cDNA preparation. cDNA is then split into “untreated” and “DASHed” libraries. Sequencing reads are processed by CellRanger and then the cell barcodes recovered from both groups are used for downstream analysis. **C** Venn diagram of cell barcodes from untreated and DASHed libraries. The shared cell barcodes are used to assess library quality. **D** Fragment analysis of untreated cDNA (top) and DASHed cDNA (bottom). x-axis shows fragment size in base pairs (bp), and y-axis shows relative fluorescence units (RFU). Red peak marks the lower marker (1 bp) and blue peak marks the upper marker (6000 bp). **E** Volcano plot of differential expression analysis of DASHed versus untreated 10X scRNA-seq datasets. Data includes 3 biological replicates of scRNA-seq libraries combined and analyzed as pseudobulk samples. x-axis is the average log2 fold change across all cells. y-axis is -log_10_ of adjusted p-value of Wilcoxon Rank Sum test based on Bonferroni correction. The cutoff for significant genes is adjusted *p*-value < 0.05 and absolute average log_2_ fold change > 1. **F** Volcano plot of differential expression analysis of DASHed versus untreated bulk RNA-seq datasets. Data includes 3 biological replicates of bulk RNA-seq libraries for either untreated or DASHed. x-axis is the average log_2_ fold change across all cells. y-axis is -log10 of adjusted p-value of Wald test based on Benjamini–Hochberg correction. The cutoff for significant genes is adjusted *p*-value < 0.05 and absolute average log2 fold change > 1

Due to the widespread use of the 10X Chromium scRNA-seq platform, and because it yields the highest library complexity in planarians, we sought to optimize the 10X protocol by selectively removing the 16S sequence. During single-cell RNAseq library preparation, reverse transcription occurs immediately after cell lysis, concomitant with barcoding, so any depletion must happen during or after this step [4]. Ribodepletion methods that remove unwanted RNA, such as RNAse-H mediated digestion, are difficult to integrate with 10X scRNA-seq because it is challenging to deplete RNA inside droplets before cDNA synthesis. Depletion of Abundant Sequences by Hybridization (DASH) is a CRISPR/Cas9-based method that effectively depletes cDNA in a sequence-specific manner, making it a promising method to eliminate the 16S transcript after barcoding [11]. Therefore, we designed 30 non-overlapping single guide RNAs (sgRNAs) tiling the entire 16S transcript. To eliminate this transcript, we reasoned that degrading 16S as early as possible after cDNA conversion would be optimal to preserve the transcriptional repertoire. We integrated DASH into the 10X Chromium workflow by only performing 10 PCR cycles after cDNA conversion, then incubating the cDNA with pooled sgRNAs complexed with Cas9. After CRISPR/Cas9 degradation, we further amplified cDNA with 10 additional PCR cycles, followed by the standard end repairing and indexing steps (Fig. 2A).

To test whether DASH could deplete 16S sequences from cDNA libraries, we first generated three 10X 3’ scRNA-seq cDNA libraries from planarian stem cells. Next, we split the cDNA libraries and generated DASH-treated (‘DASHed’) libraries for each biological replicate (Fig. 2B). By sequencing the same biological libraries before and after DASH treatment, we could benchmark the impact of DASH treatment at the single-cell level by analyzing shared cell barcodes (Fig. 2C).

To assess the efficiency of 16S depletion, we compared untreated and DASHed libraries on the Bioanalyzer. Fragment analysis showed a strong peak around 1334 bp in the untreated cDNA that was absent in the DASHed cDNA, while the rest of the profile remained comparable (Fig. 2D). Planarian 16S rRNA is predicted to be ~ 900 bp long [18, 30]. Even with the addition of 112 bp of primers from the 10X library (82 bp, poly(dT) primer + 30 bp, TSO primer), this does not account for the discrepancy in length, raising the possibility that this peak may not represent the true 16S transcript. To verify that we depleted the 16S transcript, we compiled 3 scRNA-seq replicate libraries before and after DASH treatment into a pseudobulk plot. Indeed, differential expression analysis showed that the 16S rRNA was the only downregulated gene in DASHed datasets (Fig. 2E). This specific downregulation suggests that the highest peak in the untreated library may represent 16S cDNA and that it can be efficiently depleted by DASH.

To further evaluate whether CRISPR treatment induces any off-target digestion, we performed bulk RNA-seq on samples from whole animals that were either untreated or DASHed. We sequenced three biological replicates each of *unc-22*(RNAi) and *FoxA*(RNAi) animals (Fig. 2F). The results indicate that 16S was the only downregulated gene in the DASHed datasets, suggesting that DASH treatment did not cause pervasive off-target effects.

### Overrepresentation of 16S UMIs causes aberrant cell calling

Cell calling algorithms rely on the assumption that cells have significantly more UMIs than empty droplets and ambient RNA, so they can distinguish cells from non-cells by total UMIs associated with each cell barcode [4]. We hypothesized that the high prevalence of 16S rRNA could potentially interfere with cell calling by inappropriately ‘calling’ cells that in reality are ambient RNA. To evaluate the effect of DASH treatment on cell calling, we obtained lists of cell barcodes from untreated and DASHed libraries generated by Cell Ranger for each of the 3 biological replicates (Fig. 2C). The majority of cell barcodes were shared between untreated and DASHed datasets for each replicate (Supplementary Fig. 2A). Across all 3 replicates, the untreated datasets consistently had more cell barcodes called by Cell Ranger as cells (ranging from 9 to 20% of the total) (Supplementary Fig. 2A). Of the barcodes that were unique to the untreated libraries, the vast majority (> 90%) mapped to 16S UMIs (Supplementary Fig. 2B). Barcodes shared by both untreated and DASHed libraries had 58–60% of 16S UMIs in untreated datasets, which is comparable to previously published datasets (Supplementary Fig. 2B; Fig. 1A). By contrast, the barcodes that were specific to the DASHed libraries had very low levels of 16S UMIs (< 0.2%), indicating that the depletion was thorough (Supplementary Fig. 2B). Together, these results suggest that 16S comprises a significant proportion of total UMIs in both cell and ambient RNA and misleads the cell calling process.

### DASH depletes 16S rRNA and increases mRNA and gene recovery

**Fig. 3:**
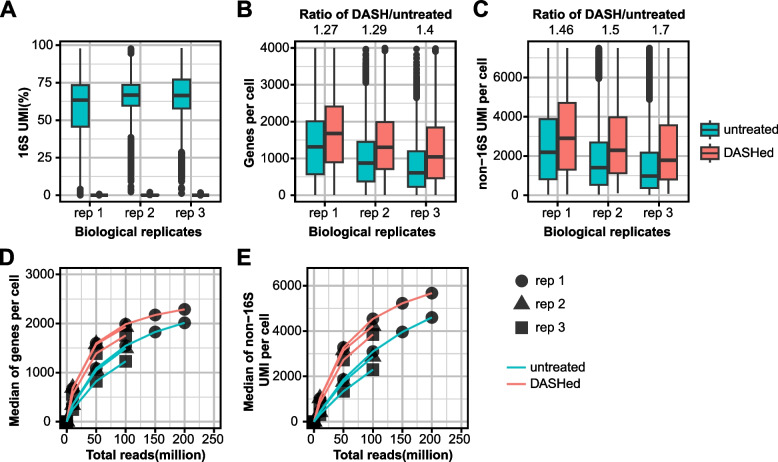
DASH depletes 16S UMIs and enhances library complexity. **A-C**, Boxplots show the percentage of 16S UMIs (**A**), numbers of genes (**B**) and non-16S UMIs (**C**) per cell in three replicates before and after DASH treatment. The medians of the ratios in DASHed versus untreated are shown on the top. Each biological replicate (rep) is downsampled to 100 million reads. **D-E** Rarefaction analysis of library complexity comparing untreated and DASHed libraries. Medians of genes (**D**) and non-16S UMI per cell (**E**) are shown. Each dot represents a downsampled replicate with indicated total reads

In general, mitochondrial and ribosomal sequences are computationally removed from downstream analysis [19, 20]. Because depletion with DASH is so specific, it may achieve the same effect as this computational processing step. Alternatively, the physical depletion of 16S at an early step in library generation could recover more sequencing information than computational removal. To assess the efficacy of 16S depletion on library complexity, we normalized untreated and DASHed scRNA-seq datasets to 100 million reads by downsampling and examined metrics of library quality. The 16S UMIs dropped from 60% to less than 0.2% in DASHed datasets, suggesting that the 16S cDNA was efficiently depleted (Fig. 3A). Moreover, the total number of genes detected per cell showed an increase ranging from 27 to 40% (Fig. 3B), and the non-16S UMIs (mostly mapped to protein-coding transcripts) increased from 46 to 70% in DASHed datasets as compared to untreated libraries (Fig. 3C). These findings suggest that DASH treatment can enrich library complexity further than computational depletion alone (Fig. 3C, D).

To investigate whether the loss of complexity in the untreated library could be overcome by simply increasing sequencing reads, we performed a rarefaction analysis. This analysis assesses library complexity by measuring how diversity increases with sequencing reads until it reaches saturation. We downsampled the datasets to 10, 50, and 100 million reads for replicates 2 and 3, and 150 and 200 million reads for replicate 1, and examined the library quality metrics of shared cell barcodes. We found that the number of genes detected per cell and non-16S UMIs were consistently higher in DASHed libraries, even at the lowest read depths (Fig. 3D-E). These results indicate that the depletion of 16S rRNA consistently enhances library complexity given the same amount of reads, beyond what can be achieved by computational ribodepletion.

### Post-CRISPR amplification is necessary and improves library complexity

**Fig. 4:**
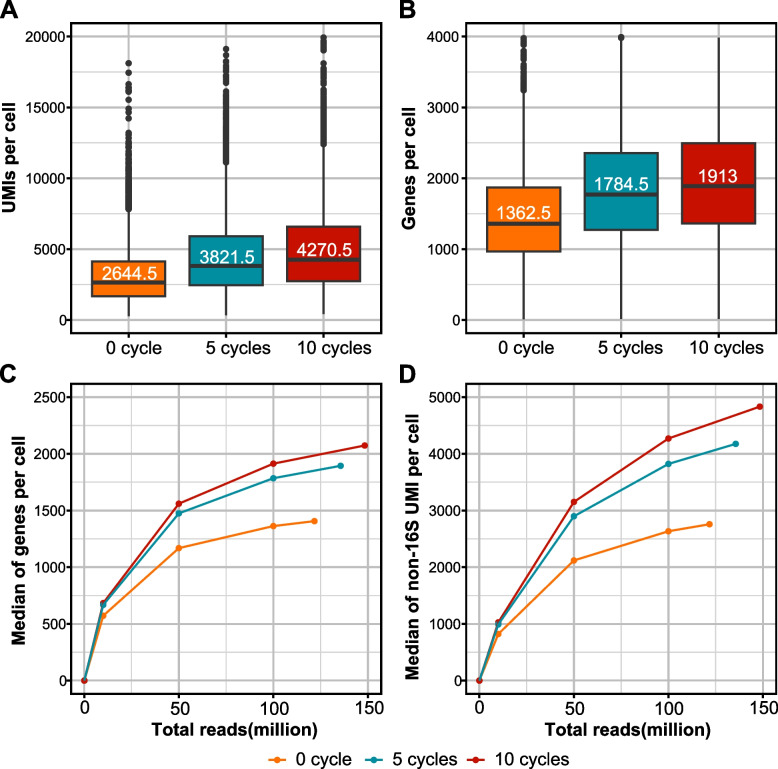
Post-CRISPR amplification increases library complexity. **A**, **B** Boxplots showing the UMIs (**A**) and genes per cell (**B**) in libraries amplified with different numbers of PCR cycles post-CRISPR. The medians are labeled in the 50th percentile in the box. **C**, **D** Rarefaction analysis of library complexity comparing the libraries amplified with different numbers of PCR cycles post-CRISPR, showing the medians of genes (**C**) and non-16S UMIs per cell (**D**). Each dot represents a downsampled replicate with indicated total reads

In scRNA-seq methods, the number of PCR cycles to amplify cDNA is adjusted based on the estimated cell numbers, tested empirically by the 10X manufacturer [4]. Over- or under-amplification leads to a decrease in library complexity. We asked if the post-CRISPR amplification is necessary and if so, what are the optimal PCR cycles that maximize library diversity without changing the overall fragment distribution. We sequenced replicate 1 without any re-amplification (0 cycles), or with 5, 10, and 15 PCR cycles of post-CRISPR amplification, and assessed library complexity by downsampling into the same total reads. cDNA amplified with 15 cycles was excluded from further sequencing because the cDNA trace changed drastically as compared to 5 and 10 cycles (Supplementary Fig. 3), suggesting that the composition of the library might have been altered by these additional PCR cycles. After sequencing, the number of UMIs and genes per cell increased with more PCR cycles (Fig. 4A-B). Similarly, the rarefaction analysis also showed that library complexity was consistently higher in the 10-cycle condition (Fig. 4C-D). This finding suggests that post-CRISPR amplification is necessary for improving library complexity, with an optimal cycle number of 10, at least for these samples.

**Fig. 5:**
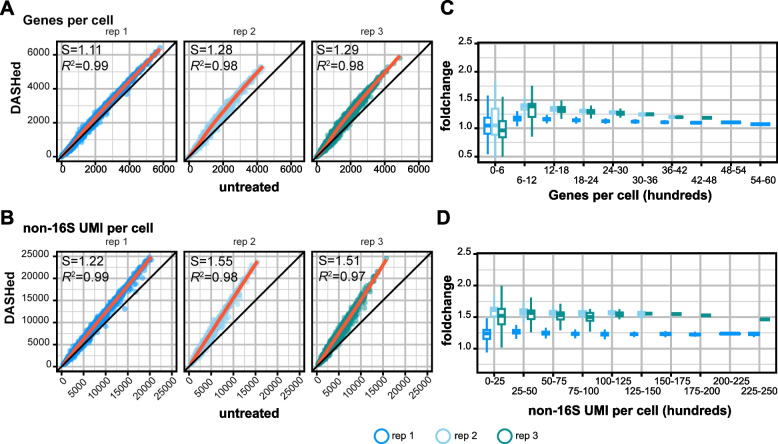
DASH treatment boosts library complexity independent of read depth. **A**, **B** Comparison of genes per cell (**A**) and mRNA per cell (**B**) between untreated and DASHed libraries. Each dot represents a cell shared by untreated and DASHed libraries. Orange lines are the linear regression models. The slope (S) of the linear regression model and its R-squared (R^2^) value are indicated in the top left. **C**, **D** Boxplots show fold changes of DASHed vs. untreated in numbers of genes (**C**) and non-16S UMIs per cell (**D**) across ranges

Since the extra PCR steps were necessary for improving library complexity, we asked whether the extra PCR was biased to enrich certain transcripts or cells that are abundant in the library. Therefore, we compared the numbers of genes and UMIs in shared cells in untreated and DASHed libraries. The number of genes and non-16S UMIs in each cell with and without DASH treatment showed strong linear correlations, suggesting the relative UMI abundance across cells was maintained after DASH treatment (R^2^ > 0.97) (Fig. 5A-B). Slopes greater than 1 in this analysis are indicative of a global increase in library complexity across cells. Moreover, we asked if these increases exist locally across different read depths. When we binned the cells into different groups based on the number of genes or UMIs, the levels of increase were consistent across different groups (Fig. 5C-D). Together, these findings show that DASH increases cell complexity in an unbiased manner.

### Downstream single-cell analysis improves after DASH

**Fig. 6:**
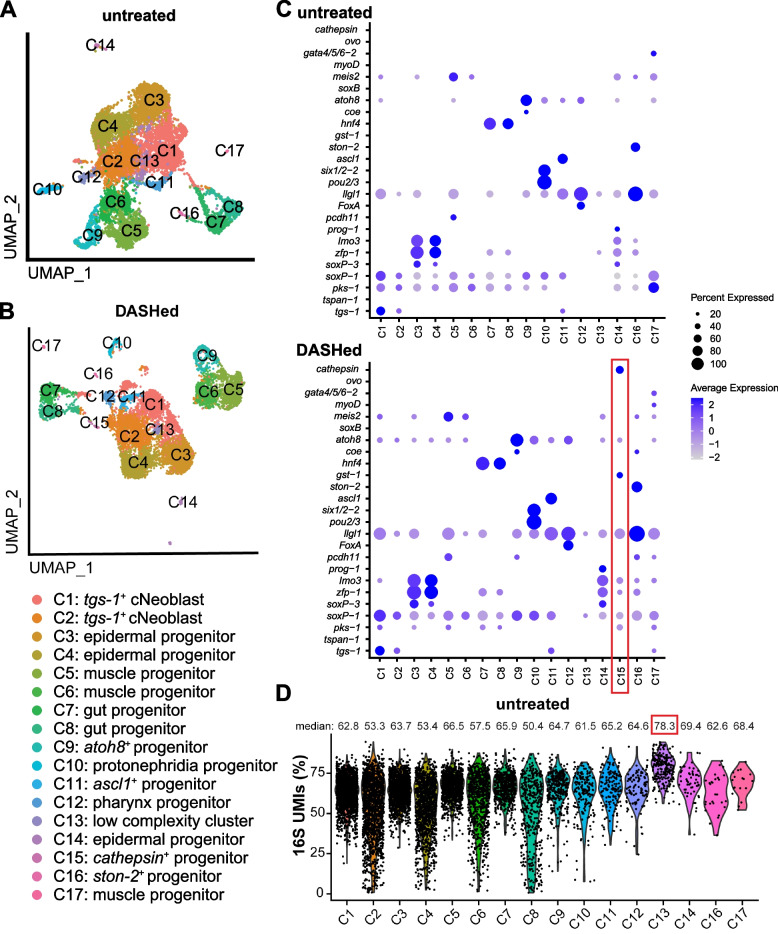
DASH treatment benefits clustering and differential expression analysis. **A**, **B** UMAP plots of untreated (**A**) and DASHed (**B**) samples. Each dot represents a single cell. Dots are color-coded by clusters. **C** Dot plots of marker gene expression across annotated clusters. The size of the dots represents the percentages of cells within each cluster that express the marker gene. The color gradient represents the expression level. Red box shows the cluster unique to the DASHed dataset. **D** Violin plot showing the percentage of 16S UMIs in each cluster, where C13 has the highest (red box). Each dot represents a single cell

**Fig. 7:**
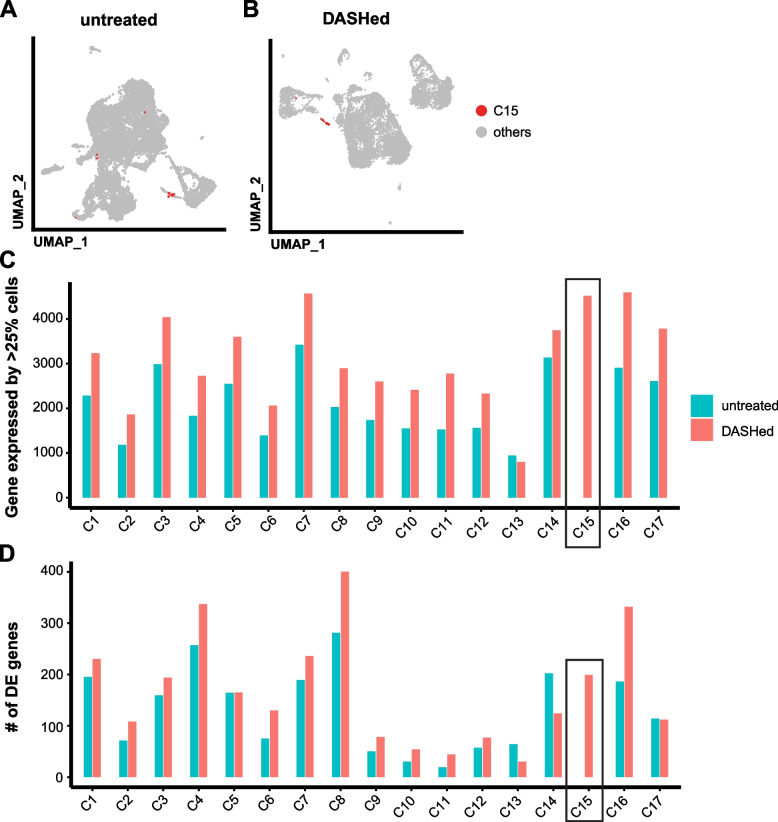
DASH treatment benefits clustering and differential expression analysis. **A**, **B** UMAP plots of untreated (**A**) and DASHed (**B**) samples. Each dot represents a single cell. Cells belonging to cluster 15 (C15) in the DASHed sample are labeled in red. **C** Bar plot showing numbers of genes that are expressed in at least 25% of cells in the same cluster. Black box indicates the cluster that is unique to the DASHed dataset. **D** Bar plot shows numbers of differentially expressed (DE) genes across clusters, tested by Wilcoxon Rank Sum test and adjusted by Bonferroni correction. Black box indicates the cluster that is unique to the DASHed dataset

By reducing contamination and ambient transcripts, the goal of DASH depletion is to reduce background and improve detection of rare cell types or more potential marker genes. To assess the benefit of DASH treatment as compared to the computational elimination of 16S, we removed 16S UMIs computationally from all libraries, and then pooled the replicates from either untreated or DASHed libraries. We used the shared nearest neighbor (SNN) method in Seurat to cluster the cells in both datasets separately (Fig. 6A-B). Clusters were annotated and aligned between two datasets based on previously described marker genes [19, 20] (Fig. 6C), except that cluster 13 could not be annotated due to the high percentage of 16S sequence (Fig. 6D). With only one exception, all other clusters were present in both groups (Fig. 7A-B). The unique cluster (C15) that appeared in the DASHed dataset represented *cathepsin*^+^ cells that were dispersed in the untreated dataset (Fig. 7A), suggesting that the clustering is more sensitive due to the increased library complexity after DASH treatment.

Differential expression analysis in scRNA-seq often selects genes expressed in at least 10–25% of the cells within the cluster to obtain robust results [31]. The number of genes expressed in at least 25% of cells of DASHed datasets (3090 genes on average) was higher than untreated datasets (2101 genes on average) across clusters, except for cluster 13, which has the highest enrichment for 16S (Fig. 7C). To identify differentially expressed (DE) genes for each cluster, we used the Wilcoxon Rank Sum test. Of the 17 clusters, 14 had more DE genes in DASHed than untreated datasets (Fig. 7D). We conclude that DASH treatment reduces the dropout rate of genes, enabling the clustering algorithm and differential expression analysis to perform better under the same parameters.

## Discussion

Sparsity of reads in single-cell sequencing is a technical challenge that arises from flooding of transcripts of housekeeping genes and ambient RNA that may swamp out detection of biologically informative genes [7, 32]. In this study, we analyzed several different scRNA-seq methodologies used in planarians and show that the single transcript for the planarian 16S rRNA accounts for 20–80% of all reads. To eliminate this contaminant, we integrated a DASH depletion step into the protocol for scRNA-seq library generation and demonstrate that depletion of the 16S sequence benefits overall library complexity. Removing the 16S transcript early during library preparation enhances the discovery of mRNAs that differentiate potential cell types, which we showed by performing a parallel analysis of untreated and depleted libraries. In conclusion, by eliminating unwanted reads, our approach improves detection of gene expression and economizes sequencing yield. Our approach is also customizable and can be adapted to any system where similar contamination is evident.

### An unusually high fraction of 16S ribosomal RNA in planarian datasets

The prevalence of mitochondrial UMIs has been widely used as quality control for identifying healthy cells in human and mouse datasets, where acceptable maximum levels range from 5–10% [33, 34]. In the planarian *S. mediterranea*, mitochondrial 16S rRNA overflows RNA-seq experiments even after poly(A) enrichment [18]*.* Following poly(A) enrichment, 16S still makes up 11–32% of total reads in bulk RNA-seq, and worsens in various single-cell methods, comprising 5–74% of total UMIs. While in most studies, the abundance of mitochondrial RNA is thought to arise from damaged cells, this is an unlikely source of 16S in planarians because most studies have used either fresh or FACS-purified live cells [18, 19, 21]. Ribosomal RNA transcribed in the nucleus is typically not polyadenylated, but reports have shown that both human and *Drosophila* mitochondrial 16S and 12S rRNA do get polyadenylated [29, 35]. Our coverage plots show that sequencing reads skew toward the 3’ end of 16S transcript, a trend resembling that of protein-coding genes. Alternatively, 3 poly(A) stretches in the middle of the 16S sequence might also contribute to its abundance, but this is unlikely because if reverse transcription initiated internally, reads corresponding to the 5’ end would be enriched over the 3’ end. Moreover, we identified one AAUAAT starting at position 808 and UUUUU starting at position 856 as potential upstream and downstream elements for poly-adenylation, respectively. Thus, we speculate that the planarian 16S might have an exceptionally long poly(A) tail, leading to significant capture in scRNA-seq, which remains to be tested experimentally. Overall, we capitalize on the overrepresentation of 16S to investigate the impact of ribodepletion on scRNA-seq.

### Alternative strategies for depleting abundant transcripts in single-cell RNA-seq

Recent advances in the ribodepletion of scRNA-seq have been shown to improve library complexity, but most of them require using new set-ups or novel reagents [8, 10]. Our DASH-mediated approach is straightforward to implement and highly customizable to any system [11]. DASH has been shown to enhance library complexity in general but has been incorporated into single-cell methods in different ways [11, 14, 16, 17]. A common workflow of single-cell transcriptomes includes these steps in order: concomitant barcoding and reverse transcription, fragmentation of cDNA, and library indexing [3, 4]. Here, we digest the 16S rRNA before the fragmentation and indexing steps of the 10X Chromium protocol. To minimize PCR bias and loss of rare transcripts, we used 10 PCR cycles, 2 cycles fewer than recommended by 10X Genomics, to amplify cDNA before CRISPR/Cas9 digestion. After CRISPR digestion, we conduct a post-CRISPR amplification to enrich cDNA diversity, resulting in less than 0.5% of total UMIs belonging to 16S. Although other single-cell total transcriptome methods include CRISPR/Cas9-mediated depletion strategies to remove ribosomal sequences, the depletion is not as complete as what we observe here (10% of reads for Smart-seq-total [14] and 34% for scDASH [16] mapped to the target genes). More recently, a technique called scCLEAN targets 255 housekeeping and ribosomal genes for removal during library preparation. Even after depletion, reads mapping to these genes still constitute 8% of the total reads [17]. The especially strong depletion that we observe here may result from several factors: (1) the contaminant in planarians is just a single transcript, and therefore easier to target and remove, (2) we used 30 sgRNAs to target one gene, compared to other approaches that use fewer sgRNAs per gene, and (3) eliminating target genes as early as possible in the library preparation may be beneficial.

### Impact of ribodepletion by DASH

While other studies have shown the overall benefit of DASH on library quality, we performed a parallel analysis of the same library before and after depletion to show the impact of DASH at single-cell resolution. This analysis reveals two key benefits of depletion. First, our data demonstrate that not depleting the 16S transcript leads to aberrant cell calling. The extra cells in untreated libraries are highly enriched in 16S UMIs, indicating that ambient RNA can falsify the cell calling process [36]. Moreover, the distribution of transcripts during initial PCR steps required for library generation is distorted by their presence. Second, depletion of 16S does not appear to introduce bias in the analysis. We conclude this because the increase in library complexity and fold change of genes increased uniformly across cells with different UMIs. Furthermore, clustering analysis showed overall agreement between untreated and DASH-treated datasets and improvement in detecting differentially expressed genes by reducing the dropout rates. These findings are important because most scRNA-seq normalization uses a “size factor”, which equalizes the cell read depth [37]. If the depletion happens disproportionately to the read depth across cells, normalization outcomes would be significantly altered by DASH.

In summary, we showcased the efficiency and robustness of DASH in scRNA-seq by demonstrating ribodepletion of 16S in planarians. The customizability of DASH will benefit any model organisms that may suffer from contamination of ambient RNA, or overabundance of irrelevant transcripts in important scRNA-seq experiments. The integration of DASH is a simple add-on to the current 10X protocol and therefore requires little expertise in developing new single-cell protocols. In addition, because the depletion can be done at any time, our protocol offers significant flexibility for sequencing after library generation.

### Limitations

The current protocol demonstrates that DASH can be successfully implemented for scRNA-seq of planarian stem cells using the 10X platform. Although we see no evidence of potential off-target effects of CRISPR digestion, we have only validated this specificity using 30 sgRNAs to degrade a single transcript and have not tested how many are sufficient. Whether DASH treatment enhances library complexity in a more complex system, such as whole animals, remains to be examined. Additionally, the number of PCR cycles used to amplify cDNA before and after DASH treatment should be tailored for optimal results.

## Conclusions

This study describes and benchmarks the ribodepletion of 16S rRNA by CRISPR-based treatment to improve scRNA-seq of planarians. Ribodepletion enhances the library complexity and performance of single-cell analysis. This demonstrates the benefit of ribodepletion in the cDNA library over in silico removal of ribosomal RNA.

## Methods

### Library quality metrics for published datasets

Parallel-fastq-dump (0.6.7) was performed to retrieve fastq files from NCBI, and the SRA accession numbers were in Supplementary Table 1. All the preprocessing and alignment in Fig. 1 use Drop-seq tools (2.3.0) except Smart-seq2 [3]. Smart-seq2 method does not have unique molecular modifiers (UMIs), so Smart-seq2 dataset was processed differently [38]. Reads of Smart-seq2 were aligned to a customized genome file containing both the chromosomal-level (Smed_chr_ref_v1) [27] and mitochondrial genomes of *Schmidtea mediterranea* (NCBI accession number: NC_022448.1) using STAR (2.7.10) [39]. Reads mapped to exon regions annotated by the SMESG gene model were extracted and segregated into a gene expression matrix. In other single-cell RNA-seq libraries, reads were aligned to the same genome file described above using Drop-seq tools[3]. Cell barcodes and unique molecular identifiers (UMIs) were extracted and tagged to the reads in BAM format and further segregated into a gene expression matrix. To calculate library quality metrics, ‘CreateSeuratObject’ function of Seurat (4.3.0) in R(4.2.0) was used to import the count matrix from Drop-seq tools or Smart-seq2 pipeline, which calculates total UMI counts and the number of genes expressed associated with each cell barcode [31]. Further, custom R codes were used to extract 16S UMIs and non-16S UMIs (total UMIs—16S UMIs). The workflow was adapted from TAR-scRNA-seq and compiled in snakemake (7.18.2) [40].

### Worm care

*Schmidtea mediterranea* asexual clonal line CIW4 was raised in a recirculating water system supplemented with water containing Montjuïc salts [41, 42]. Animals were fed with beef liver and cleaned once a week. Animals were transferred to static culture containing 50 µg/mL gentamicin for at least a week prior to use.

### Cell sorting

For each biological replicate, 10 animals were dissociated into single-cell suspensions by dicing in CMFB buffer [calcium-magnesium-free solution with 1% BSA (400 mg/L NaH_2_PO_4_, 800 mg/L NaCl, 1200 mg/L KCl, 800 mg/L NaHCO_3_, 240 mg/L glucose, 1% BSA, 15 mM HEPES, pH7.3)] and nutating for 2 h at room temperature. Cells were centrifuged at 500 g for 5 min, resuspended, and filtered through a 30 µm cell strainer (BD Biosciences, 340,628) to remove debris. The concentration of filtered cells was calculated using a TC20 automated cell counter (Bio-Rad). After centrifugation, cell concentration was adjusted to 100,000 cells/mL with staining buffer [CMFB containing DRAQ5 (5 µM) and Calcein-AM (0.4 µM)] and nutated at room temperature for 5 min. X1 cells were gated for vital 4N cells (DRAQ5^+^ Calcein-AM^+^) on a Sony MA900 Cell Sorter. 100,000 cells were sorted and diluted to a concentration of 1000 cells/µL for subsequent library preparation.

### sgRNA design and in vitro transcription

The detailed protocol for DASH is in the Supplementary Information. The 16S ribosomal RNA sequence was retrieved from the mitochondrial genome of *Schmidtea mediterranea* (NCBI accession number: NC_022448.1) and uploaded to IDT’s Alt-R Custom Cas9 crRNA Design Tool. 30 non-overlapping seed regions were selected from the output of the Design Tool to ensure complete digestion of the entire 905 bp transcript. The primer sequences are listed in Supplementary Information. To synthesize T7-flanking templates for sgRNA, PCR reactions were assembled following the Phusion High-Fidelity (NEB) protocol with final concentrations of primers: one of the sgRNA primers (0.2 µM), T7RevLong (0.2 µM), T7FwdAmp (1 µM), T7RevAmp (1 µM). PCR reactions were carried out as follows: 98 °C 30 s, repeating the steps of 98 °C for 10 s, 51 °C for 10 s, 72 °C for 10 s 30 times, and then 72 °C for 2 min. PCR products were run on agarose gels to determine whether primers remained. If they did, the PCR products were gel purified. To synthesize sgRNA, the concentration of templates of each sgRNA was measured by nanodrop and pooled equivalently. In vitro transcription reactions were assembled as followed: sgRNA templates (4 µg), 10X transcription buffer [0.1 M MgCl_2_, 0.4 M Tris (pH 8.0), 0.1 M DTT, 20 mM spermidine] (10µL), 25 mM rNTPs (Promega) (8µL), T7 polymerase (generated in-house) (2µL), TIPP (NEB) (2µL), rRNAsin (Promega) (1µL) and nuclease-free water (adjusting the total volume to 100 µL). In vitro transcription reactions were incubated at 37 °C overnight. The next day, 2 µL RQ1 DNase (Promega) was added to remove templates and incubated at 37 °C for 20 min. To precipitate the sgRNAs, 250 µL ice-cold 100% ethanol was added to each reaction and incubated at -20 °C for 1 h. sgRNAs were pelleted by centrifugation at 4°C for 2 min at 17,000 g, and the supernatant was removed. To wash, 250 µL 70% ice-cold ethanol was added, followed by centrifugation for 2 min at 17,000 g twice. The sgRNAs were resuspended in 10 µL nuclease-free water.

### 10X single-cell library preparation and DASH

Sorted cells were counted and checked viability on a Countess 3 Automated Cell Counter (ThermoFisher) with Trypan blue (0.4%) staining. Sorted cells showing > 85% viability were used for 10X single-cell library preparation. To aim for recovery of 5000 cells after sequencing, 8250 cells were loaded onto the 10X Genomics Chromium Controller for subsequent library preparation using Chromium Next GEM Single Cell 3ʹ Reagent Kit v3.1. Samples were amplified with 10 PCR cycles with cDNA primers (R1 + TSO) after clean-up. For DASH, 29 µL CRISPR master mix [NEBuffer 3.1 (3µL), 20 μM sgRNAs (1µL, final 0.66 μM), 1 µM Cas9 Nuclease (NEB, M0386S) (2µL, 66 nM), nuclease-free water (23 µL)] was mixed and pre-incubated at 37°C for 10 min. Then, 1µL (1–10 ng/µL) of cDNA was added to the master mix and incubated at 37°C overnight. After CRISPR treatment, the cDNA was cleaned up and eluted into 15 µL using AMPure beads (Beckman) following the manufacturer’s protocol. Then, the cDNA was diluted to 30 µL and amplified with cDNA primers (R1 + TSO) again with 10 cycles unless otherwise specified in this study. After PCR amplification, the cDNA was processed as specified in the 10X Genomics protocol for enzymatic fragmentation and indexing. Three biological replicates were used in this study. Libraries were pooled and sequenced using the NextSeq 2000 platform (Illumina). To assess whether 16S cDNA was removed, we ran individual samples on the Fragment Analyzer (Agilent).

### Bulk RNA-seq preparation and analysis

Bulk RNA-seq libraries were generated from either *unc-22*(RNAi) or *FoxA*(RNAi) animals 14 days after the last RNAi feeding. RNAi was conducted as previously described [43]. Three biological replicates were generated from 5 whole animals for each RNAi condition. RNA was isolated using Trizol, and cDNA was generated using NEBNext® Ultra™ II Directional RNA Library Prep Kit for Illumina®. For DASH treatment, 1 µL of cDNA was mixed with CRISPR master mix to deplete 16S. Untreated and DASHed cDNA were then end-prepped and ligated to adaptors for indexing PCR. All libraries were pooled together and sequenced by HiSeq X Ten sequencer (2 × 150 bp). For the analysis, the sequencing reads were mapped to aligned to the same genome file as described above using STAR (2.7.10) [39], and reads mapped to exon regions annotated by SMESG gene model were extracted and segregated into a gene expression matrix. DESeq2 (3.17) was used to conduct differential expression analysis in R(4.2.0) [44].

### Single-cell analysis for untreated and DASHed libraries

Figure 2B demonstrates the analysis workflow. The untreated and DASHed libraries from three biological replicates were processed by Cell Ranger (6.1.2) [4]. The lists of cell barcodes were retrieved from both libraries and split into “untreated-specific”, “shared” and “DASHed-specific” categories (Supplementary Fig. 3). The percentage of 16S UMIs was calculated for each category. For the rest of the analysis, only “shared” cells were used.

For rarefaction analysis, we downsampled the datasets into 10, 50, and 100 million reads for replicate pairs 2 and 3, and 150 and 200 million reads for replicate pair 1. Downsampled datasets were processed independently in CellRanger.

For clustering analysis, the shared cells were further selected if the cells had (1) more than 200 genes and (2) *piwi-1* expression ≥ 2.5 [log_10_(UMI-per-10,000 + 1)] to remove cells with low complexity and non-stem cells. If a cell didn’t meet the criteria in the untreated dataset, the same cells would be also eliminated in the DASHed dataset. Subsequently, all three replicates of either untreated or DASHed libraries were pooled separately. The libraries were normalized and scaled in Seurat and then integrated by Harmony (0.1.1) [45]. The first fifty Harmony coordinates were used to calculate UMAP embedding. The clustering used the first twenty Harmony coordinates with a resolution of 0.5 for the FindClusters function, which applies shared nearest neighbor (SNN) method, resulting in 16 clusters in untreated and 17 clusters in DASHed datasets [31]. The list of markers from Zeng et al. (2018) was used to manually annotate and align the clusters [20]. To detect differentially expressed genes, Wilcoxon Rank Sum adjusted by Bonferroni correction was used to compare untreated and DASHed samples (Fig. 2E) or across clusters (Fig. 7C, D). Differentially expressed genes (1) had an adjusted *p*-value < 0.05, (2) were expressed by at least 25% of cells within the clusters and (3) log2 fold change > 0.25 compared to other clusters.

### Supplementary Information

**Additional file 1: Supplementary figure 1.** 16S reads are enriched at the 3’ end of the locus. **Supplementary figure 2.** Overrepresentation of 16S UMI causes aberrant cell calling. **Supplementary figure 3**. 10 PCR cycles post-CRISPR is optimal. **Supplementary Table 1.** Quality metrics of planarian single cell datasets.

## Data Availability

All data is available upon request. scRNA-seq data have been deposited into NCBI with Accession number GSE231548. Code for generating the figures can be found on github https://github.com/kw572/DASH_figures.
