# Supplementary material for: CRISPR/Cas9-based depletion of 16S ribosomal RNA improves library complexity of single-cell RNA-sequencing in planarians
Source: BMC Genomics. 2023 Oct 20;24:625. doi: 10.1186/s12864-023-09724-4 (PMC10588366; doi:10.1186/s12864-023-09724-4)
Supplement: Supplementary file 1 — Additional file 1: Supplementary figure 1. 16S reads are enriched at the 3’ end of the locus. Supplementary figure 2. Overrepresentation of 16S UMI causes aberrant cell calling. Supplementary figure 3. 10 PCR cycles post-CRISPR is optimal. Supplementary Table 1. Quality metrics of planarian single cell datasets. [file 12864_2023_9724_MOESM1_ESM.pdf]

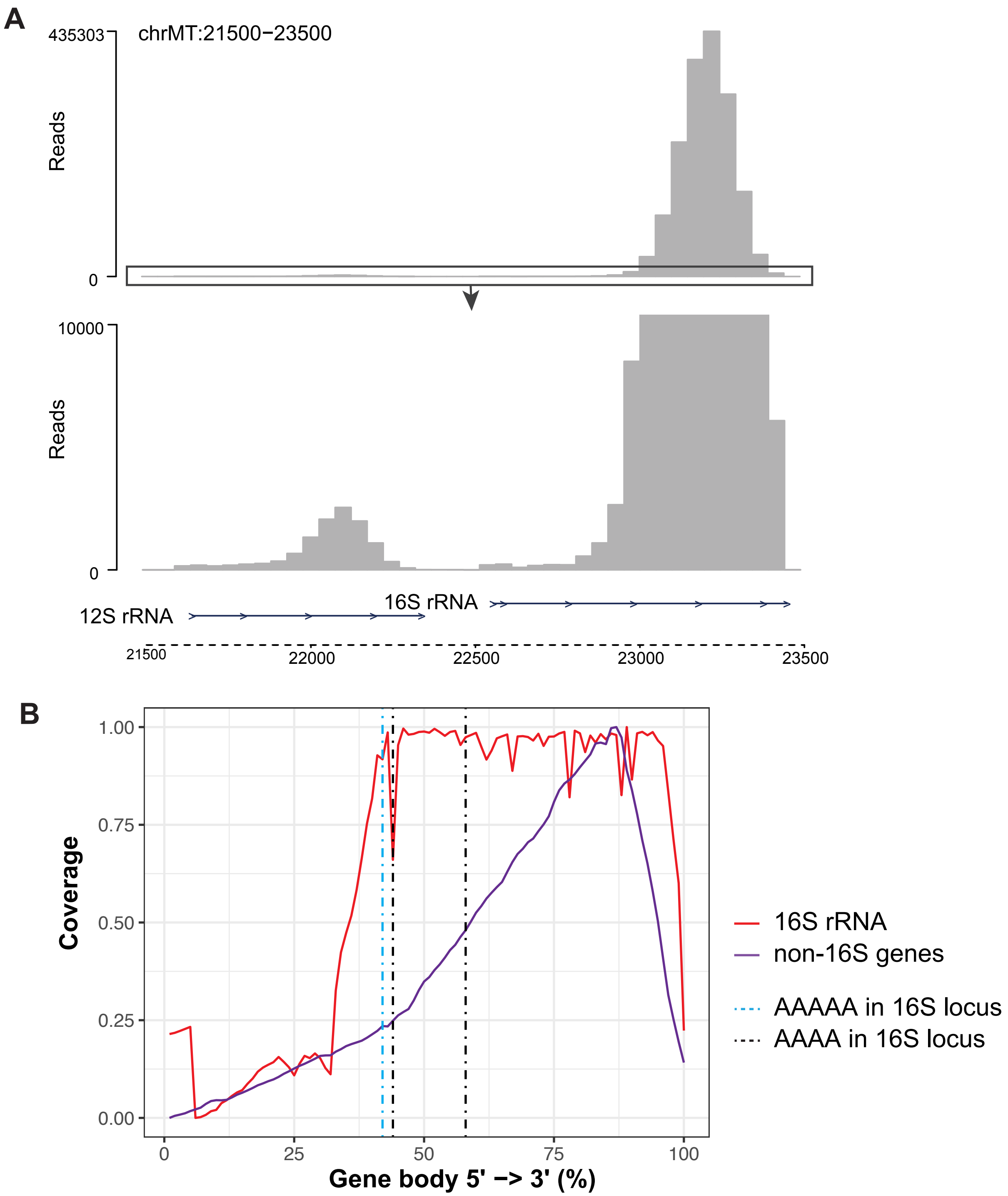

Supplementary figure 1. 16S reads are enriched at the 3' end of the locus.
A, Genomic tracks showing the raw read depth surrounding 12S and 16S rRNA loci, The bottom track shows 0-10000 to highlight the 12S locus. Arrows show the orientations of gene loci.
B, Gene body coverage plot showing the coverage of 16S rRNA locus (red) and average coverage of all non-16S gene loci (purple) in planarians. The coverage is retrieved from the untreated library, replicate 2. x-axis represents the percentage of the gene body, from 0% (5' end) to 100% (3' end). y-axis represents the relative coverage of reads that map to each part of the gene body. Dashed lines indicate the positions of A stretches in 16S locus.

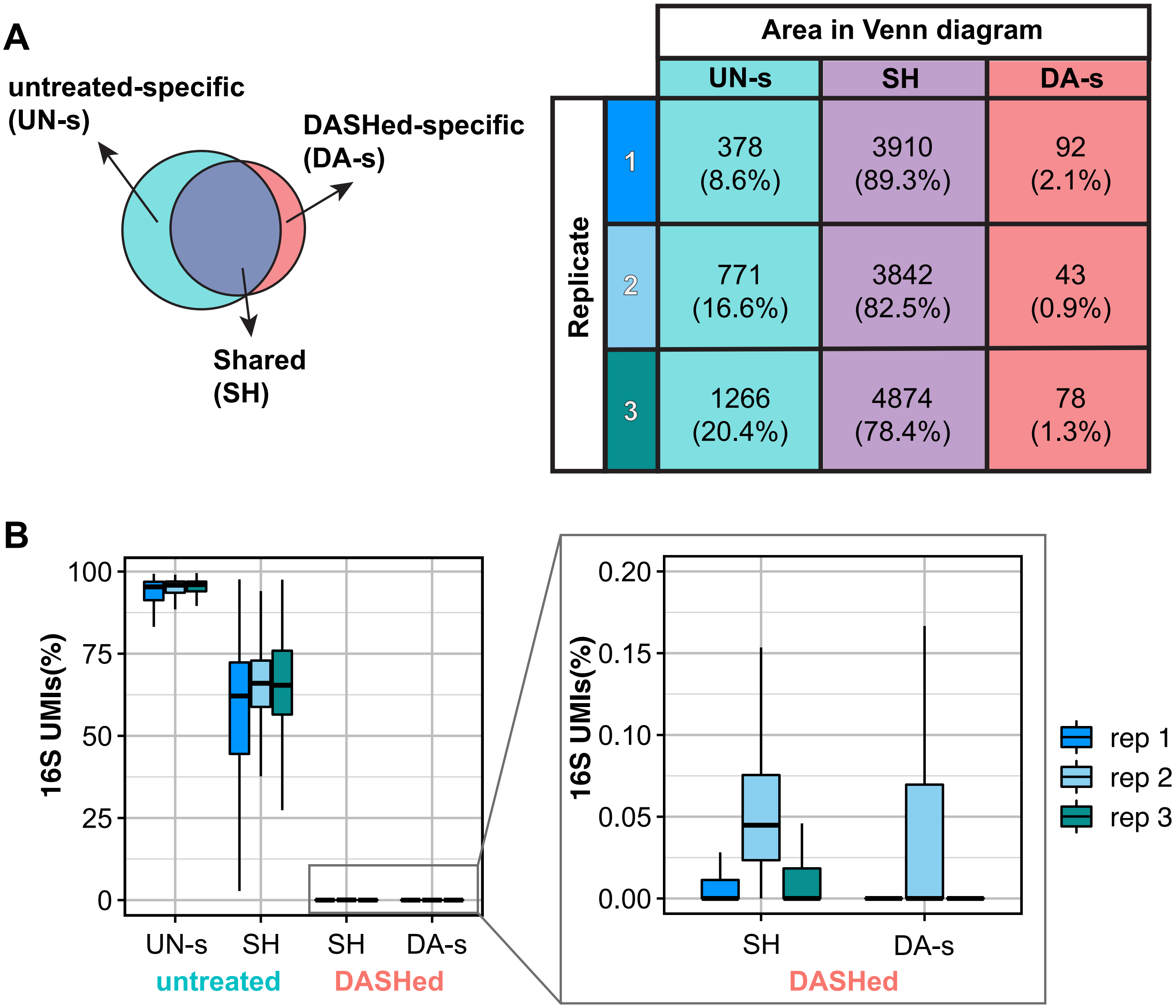

Replicate	UN-s	SH	DA-s
1	378 (8.6%)	3910 (89.3%)	92 (2.1%)
2	771 (16.6%)	3842 (82.5%)	43 (0.9%)
3	1266 (20.4%)	4874 (78.4%)	78 (1.3%)

(Column header: Area in Venn diagram)

Supplementary figure 2. Overrepresentation of 16S UMI causes aberrant cell calling.
A, Venn diagrams show the cell barcodes shared or specific to “untreated” or “DASHed” across biological replicates. Table shows the number of cell barcodes in each area and replicate. The percentages show the proportion of the numbers over the total cell numbers within the replicate.
B, Box plots depict the percentage of 16S UMI from the cells of indicated area. Enlarged box plots from B show cells from “DASHed” libraries.

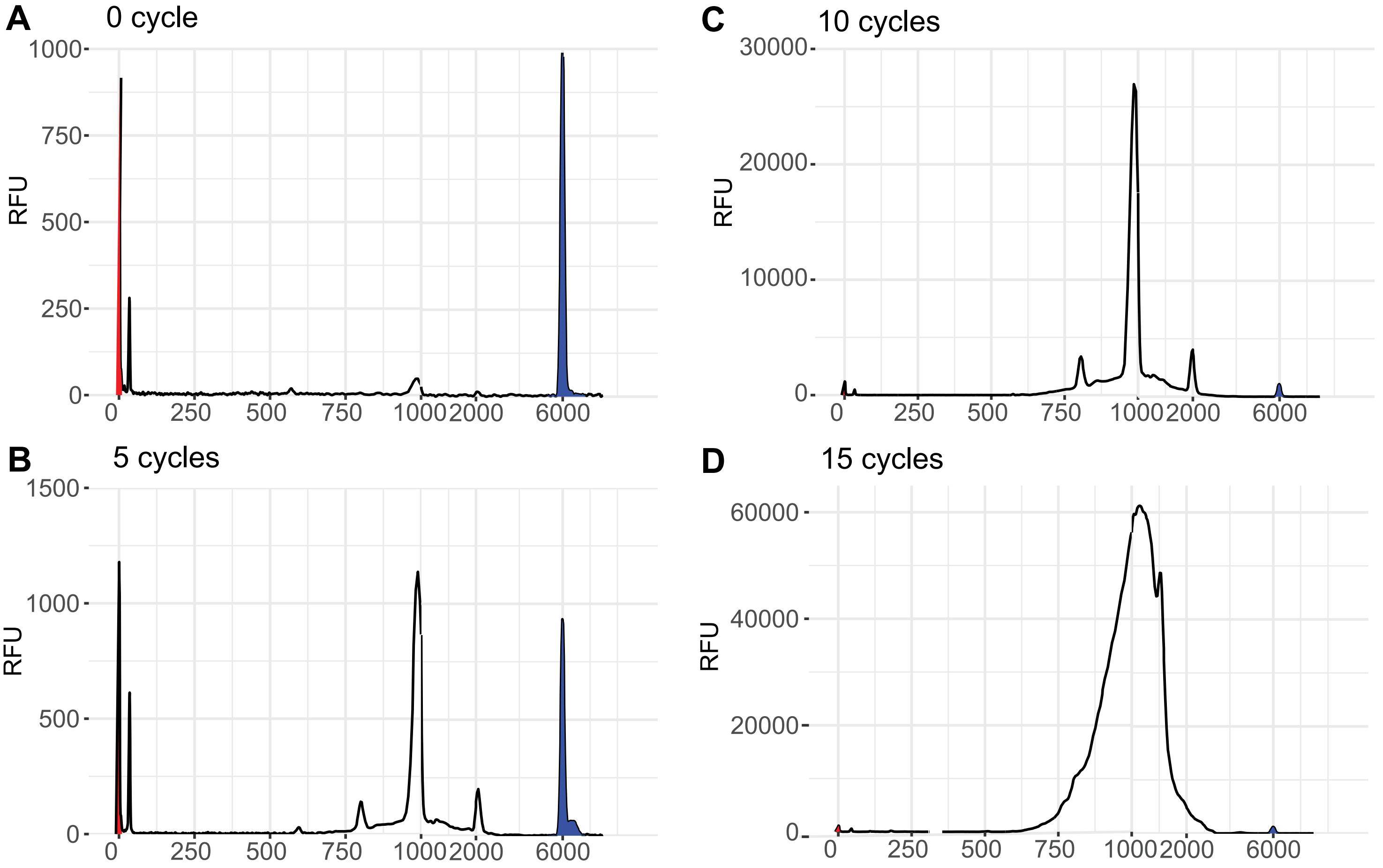

Supplementary figure 3. 10 PCR cycles post-CRISPR is optimal.
Fragment analysis of cDNA with 0 (A), 5 (B), 10 (C) and 15 (D) cycles post-CRISPR.
x-axis shows fragment size in base pairs (bp), and y-axis is relative fluorescence units (RFU). Red peak marks the lower marker (1bp) and blue peak marks the upper marker (6000bp).

Supplementary Table 1: Quality metrics of planarian single cell datasets

dataset	Median of genes	Median of UMIs	Mean of genes	Mean of UMIs	16S%	12S%	mito%	Total Reads	Uniquely mapped reads %	% of reads mapped to multiple loci	% of reads unmapped: too short	SRA files
ACME	313.5	654.5	421.75	898.88	25.24%	0.50%	1.32%	99,760,985	21.90%	21.10%	53.94%	SRR11768230
Fincher.1	627.5	1447	870.32	2418.39	21.45%	0%	0.67%	21,927,812	65.08%	10.24%	21.13%	SRR6829380
Fincher.2	645.5	1588	868.04	2473.85	25.36%	0%	0.78%	50,022,789	60.08%	9.50%	25.55%	SRR6829381
Fincher.3	657.5	1486	872.37	2433.80	25.56%	0%	0.89%	99,120,749	63.57%	9.85%	21.94%	SRR6829382
Fincher.4	936	2381	1031.81	3007.32	26.64%	0.03%	1.14%	487,831,951	69.84%	10.95%	14.87%	SRR6829389;SRR6829390;SRR6829391;SRR6829392
Fincher.5	886	2373	972.57	2866.42	32.87%	0.04%	1.61%	332,546,131	68.65%	9.71%	18.33%	SRR6829395;SRR6829396;SRR6829397
Plass	349	1365	456.76	1801.93	52.05%	0.38%	4.44%	152,686,345	75.57%	7.85%	14.06%	SRR6020457
Scimone	1648	14338	1658.40	14723.31	74.37%	0.74%	2.24%	246,559,964	74.28%	7.07%	16.63%	SRR15016138
SmartSeq2	1623	706915	1821.77	811164.43	39.18%	0.12%	5%	1,473,623	32.64%	35.20%	27.18%	GEO:GSE79866
SplitSeq.1	406	647	475.79	807.41	5.15%	1.26%	0.54%	52,438,770	47.45%	39.08%	4.86%	SRR11276863
SplitSeq.2	891.5	1524	1011.77	1821.92	4.54%	1.30%	0.87%	96,156,650	48.35%	39.11%	5.29%	SRR11276865
SplitSeq.3	348	613.5	433.34	811.40	8.36%	3.58%	1.41%	76,997,859	44.66%	43.20%	4.96%	SRR11276867
Zeng	2481	14358	2463.81	15166.24	60.58%	0.26%	3.25%	562,100,456	81.59%	4.62%	13.69%	SRR6363908

Single-cell DASH with 10X single-cell sequencing

Introduction

DASH effectively depletes unwanted cDNA sequences in bulk RNA-seq libraries with minimal input (1ng) by CRISPR-Cas9 cleavage. DASH has also been applied to several single-cell RNA-seq protocols[1–3]. Here, we demonstrate DASH is also adaptable to 10X single-cell sequencing pipeline and effectively depletes planarian 16S ribosomal RNA that dominates planarian single-cell transcriptomes. This depletion of 16S rRNA saves cost, enhances library complexity, and does not compromise downstream analysis.

Prepare sgRNAs[4]

1. The gRNAs are designed to target 16S in a non-overlapping manner by IDT guide RNA design tool.

T7RevLong	AAAAAAAGCACCGACTCGGTGCCACTTTTTCAAGTTGATAACGGACTAGCCTTATTTAA ACTTGCTATGCTGTTTCCAGCATAGCTCTTA
T7FwdAmp	TAATACGACTCACTATAG
T7RevAmp	AAAAAAAGCACCGACTCGGTGC
16S_1	TAATACGACTCACTATAGATATTTTCCTTTTTGTATTAGTTTAAGAGCTATGCTGGAAAC
16S_2	TAATACGACTCACTATAGCTTTTTGTATTACGGTTTGTGTTTAAGAGCTATGCTGGAAAC
16S_3	TAATACGACTCACTATAGTATGTTTTTATTCTCTCGAAGTTTAAGAGCTATGCTGGAAAC
16S_4	TAATACGACTCACTATAGGTGTTTTGTTTTATTTTCTTGTTTAAGAGCTATGCTGGAAAC
16S_5	TAATACGACTCACTATAGTGATAGTAGTGATATGTTTTGTTTAAGAGCTATGCTGGAAAC
16S_6	TAATACGACTCACTATAGTCTAGTTTTTAATTGTCTTTGTTTAAGAGCTATGCTGGAAAC
16S_7	TAATACGACTCACTATAGTAGTCGTTTGTTTGCTTATAGTTTAAGAGCTATGCTGGAAAC
16S_8	TAATACGACTCACTATAGATTTAACTTATCAGTGAGCAGTTTAAGAGCTATGCTGGAAAC
16S_9	TAATACGACTCACTATAGGCAGTACTTTGACTGTACGAGTTTAAGAGCTATGCTGGAAA C
16S_10	TAATACGACTCACTATAGTAATTCTAGAATTGTTTGAAGTTTAAGAGCTATGCTGGAAAC
16S_11	TAATACGACTCACTATAGAAAGTATAAATTAAAAAACAGTTTAAGAGCTATGCTGGAAAC
16S_12	TAATACGACTCACTATAGAGATACAGTTTGTTATTTCAGTTTAAGAGCTATGCTGGAAA
16S_13	TAATACGACTCACTATAGCTAGAGAGTTTTTAACTTAGGTTTAAGAGCTATGCTGGAAAC
16S_14	TAATACGACTCACTATAGTTCTTACTTTAGTTATTTGTGTTTAAGAGCTATGCTGGAAAC
16S_15	TAATACGACTCACTATAGTTAGTTATTTGTTGGGGTAAGTTTAAGAGCTATGCTGGAAAC
16S_16	TAATACGACTCACTATAGATTACATTATGAACTTCCTTGTTTAAGAGCTATGCTGGAAAC
16S_17	TAATACGACTCACTATAGTGAACTTCCTTAGGGATAACGTTTAAGAGCTATGCTGGAAA C

16S_18	TAATACGACTCACTATAGCTATATCCCTGTTATCCCTAGTTTAAGAGCTATGCTGGAAAC
16S_19	TAATACGACTCACTATAGATAACAGGGATATAGAATCTGTTTAAGAGCTATGCTGGAAAC
16S_20	TAATACGACTCACTATAGACAGGGATATAGAATCTTGGGTTTAAGAGCTATGCTGGAAAC
16S_21	TAATACGACTCACTATAGTTAACTACAATTCAACATCGGTTTAAGAGCTATGCTGGAAAC
16S_22	TAATACGACTCACTATAGGTTGAATTGTAGTTAAAGATGTTTAAGAGCTATGCTGGAAAC
16S_23	TAATACGACTCACTATAGGTAGTTAAAGATAGGTGTAGGTTTAAGAGCTATGCTGGAAAC
16S_24	TAATACGACTCACTATAGGTCGAACAGACTAAAAGTAAGTTTAAGAGCTATGCTGGAAAC
16S_25	TAATACGACTCACTATAGAGTTTAGACCGATGTGAATCGTTTAAGAGCTATGCTGGAAAC
16S_26	TAATACGACTCACTATAGTAGACCGATGTGAATCAGGTGTTTAAGAGCTATGCTGGAAAC
16S_27	TAATACGACTCACTATAGAAAACCAACCTGATTCACATGTTTAAGAGCTATGCTGGAAAC
16S_28	TAATACGACTCACTATAGCGTACAATGGACAGAAATTCGTTTAAGAGCTATGCTGGAAAC
16S_29	TAATACGACTCACTATAGGTCCATTGTACGAAAGGAATGTTTAAGAGCTATGCTGGAAAC
16S_30	TAATACGACTCACTATAGATCCAATTCCTTTCGTACAAGTTTAAGAGCTATGCTGGAAAC

2. To synthesize gRNA templates, make PCR reactions as below:

Component	Volume
Nuclease-free Water	10.6 µl
5x Phusion HF buffer	4 µl
10mM dNTPs	0.4 µl
*16S_number	0.4 µl
T7RevLong	0.4 µl
T7FwdAmp	2 µl
T7RevAmp	2 µl
Phusion HF DNA polymerase	0.2 µl

Total	20 μl

* 16S_number refers to each oligo name starting with 16S

3. Run PCR as below:

Step1	98℃	30 sec
Step2	98℃	10 sec
Step3	51℃	10 sec
Step4	72℃	10 sec<br>*30 times step 2-4
Step5	72℃	2 min
Step6	12℃	hold

4. Run electrophoresis to make sure the PCR product is a single band without excessive primers
5. No PCR cleanup necessary. Otherwise, do gel purification.
6. Measure the concentration by nanodrop
7. Pool all the DNA templates equivalently
8. In vitro transcription
   Example:

sgRNA templates	4 μg
10X buffer	10 μl
rNTP 25mM	8 μl
T7 polymerase	2 μl
TIPP	2 μl
rRNAsin	1 μl
Nuclease-free water	Bring to 100 μl

9. Add 1uL RQ1 RNAse-free DNAse, incubate RT for 20 minutes
10. Add 250uL 100% Ethanol
11. Incubate at -20℃ for 1 hour (can go overnight here)
12. Spin at max speed for 20 minutes at 4℃
13. Remove solvent from the pellet, and add 1mL ice-cold 70% ethanol
14. Vortex briefly and spin at max speed for 2 minutes at 4℃

15. Remove solvent from the pellet, and add 1mL ice-cold 70% ethanol
16. Vortex briefly and spin at max speed for 2 minutes at 4℃
17. Remove the solvent, and place the sample at 37℃ with the lid open until dry
18. Dissolve in the desired volume of the appropriate buffer. Suspending in 1/10th of the initial transcription reaction volume should yield around 2mg/mL sgRNAs

Prepare 10X single cell libraries[5]

1. Dissociate desired tissues into cell suspension
2. Stain and filter for FACS sorting
3. Collect targeted numbers of cells according to Chromium Single Cell 3' Reagent Kits User Guide (v3.1 Chemistry)
4. Resuspend into 1000 cells/µl in buffer recommended by Chromium Single Cell 3' Reagent Kits User Guide (v3.1 Chemistry). In this study, we use CMFB buffer [calcium-magnesium-free solution with 1% BSA (400mg/L NaH_2PO_4, 800 mg/L NaCl, 1200 mg/L KCl, 800 mg/L $NaHCO_3$, 240 mg/L glucose, 1% BSA, 15 mM HEPES, pH7.3)]
5. Follow 10X chromium protocol and target 5000 cells/library until finishing Step 2 in Chromium Single Cell 3' Reagent Kits User Guide (v3.1 Chemistry)

DASH treatment[6]

1. After Step 2 in Chromium Single Cell 3' Reagent Kits User Guide (v3.1 Chemistry), the yield of cDNA will be 1-10ng/µl (from 3000-5000 X1 cells)
2. Assemble CRISPR master mix as below:

Component	Volume	Final
Nuclease-free Water	23 µl	
NEBuffer 3.1	3 µl	1X
20 µM sgRNA	1 µl	0.66 µM
1 µM Cas9 Nuclease (M0386S)	2 µl	66 nM
Total	29 µl	

3. Pre-incubate for 10 min at 37℃
4. Add 1 µl of cDNA into CRISPR master mix
5. Incubate at 37℃ overnight

cDNA purification

1. Mix CRISPR reaction with 1.8X Ampure Beads (Beckman) with 10 times pipetting
2. Let stand for 5 min at room temperature
3. Place the tube on a magnetic stand for 3 min to separate the beads from the solution
4. Wash the beads with 80% freshly prepared EtOH 30 sec twice on the magnetic stand
5. Air dry for 3 min on the magnetic stand
6. Remove tubes from the magnetic stand and resuspend the beads with 15 µl Nuclease-free Water
7. Stand for 5 min at room temperature
8. Place the tube on a magnetic stand for 3 min to separate the beads from the solution
9. Transfer the solution into a new tube

Library preparation

1. Repeat Step 2.2 in Chromium Single Cell 3' Reagent Kits User Guide (v3.1 Chemistry) with 10 PCR cycles.
2. QC check on fragment analyzer
3. Proceed library preparation in Chromium Single Cell 3' Reagent Kits User Guide (v3.1 Chemistry)

Expected results from fragment analyzer:
Before DASH:

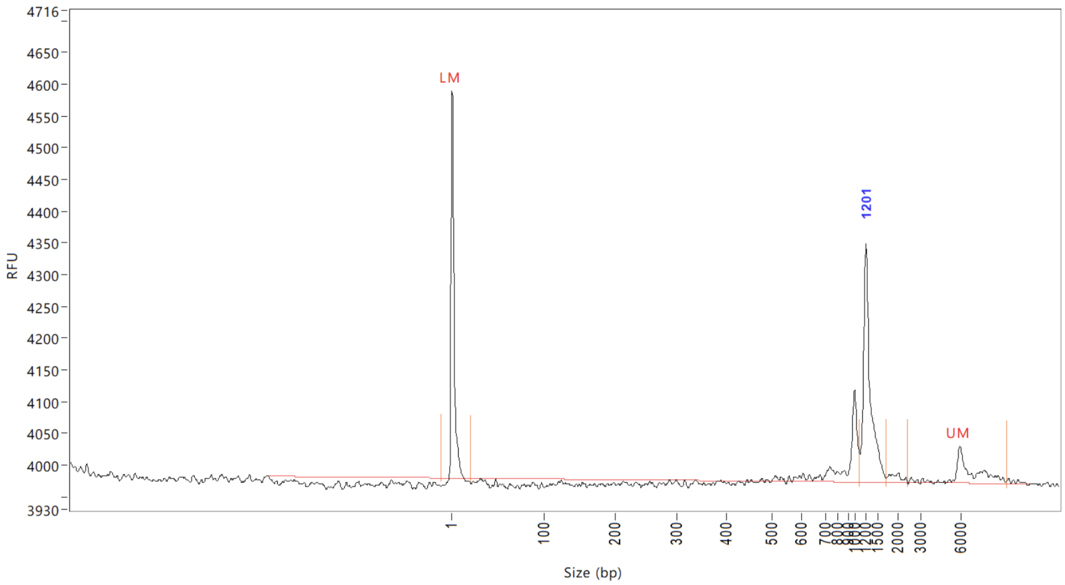

*Peak around 1200-1300 comes from 16S rRNA

After DASH:

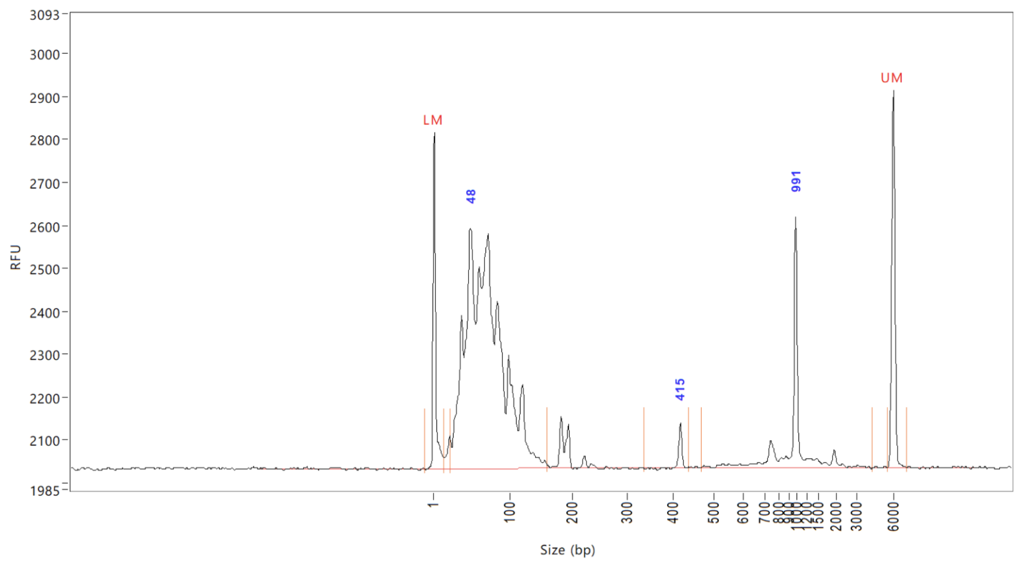

*The 16S rRNA peak should be gone, and the breakdown of 16S rRNA will be below 200 bp

After DASH+10 PCRcycle:

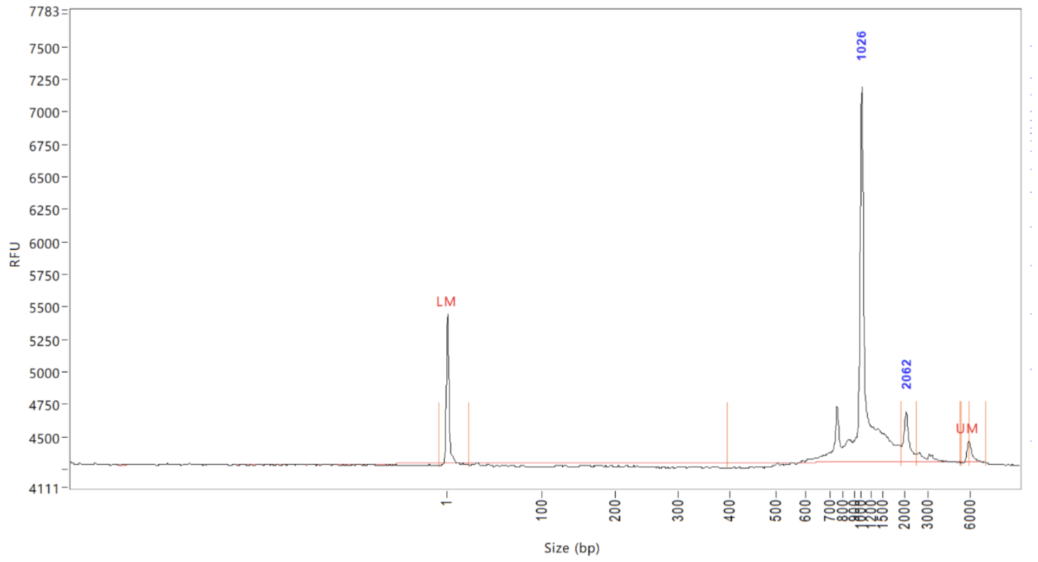

*Additional round of Step2.2 can get rid of 16S rRNA breakdown

After fragmentation and indexing:

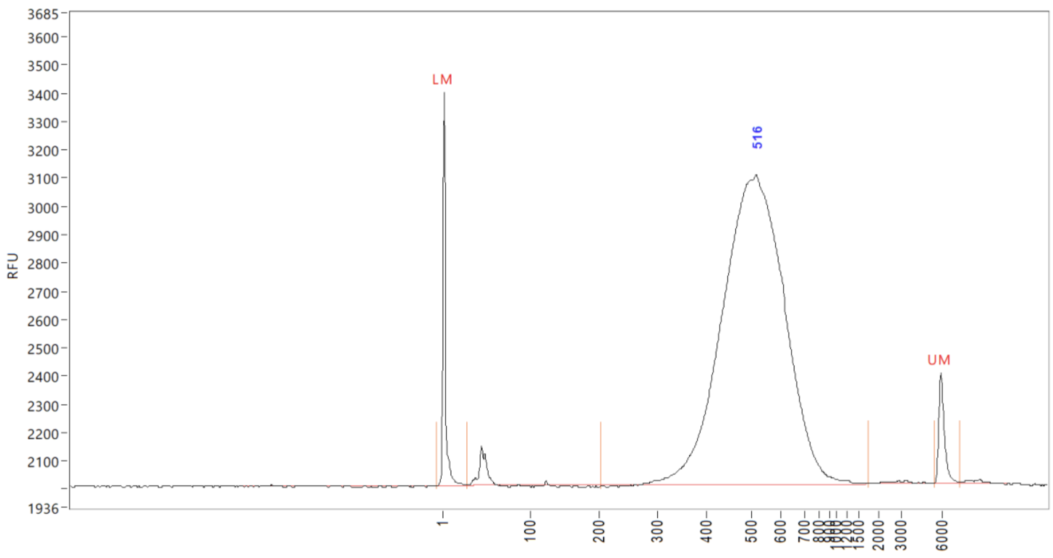

RFU
LM
516
UM
3685
3600
3500
3400
3300
3200
3100
3000
2900
2800
2700
2600
2500
2400
2300
2200
2100
1936
1
100
200
300
400
500
600
700
2000
3000
6000

Reference

1. Pandey, A. C. *et al.* A CRISPR/Cas9-based enhancement of high-throughput single-cell transcriptomics. *bioRxiv* 2022.09.06.506867 (2022) doi:10.1101/2022.09.06.506867.
2. Isakova, A., Neff, N. & Quake, S. R. Single-cell quantification of a broad RNA spectrum reveals unique noncoding patterns associated with cell types and states. *Proc. Natl. Acad. Sci. U. S. A.* **118**, (2021).
3. Loi, D. S. C., Yu, L. & Wu, A. R. Effective ribosomal RNA depletion for single-cell total RNA-seq by scDASH. *PeerJ* **9**, e10717 (2021).
4. Dewitt, M. In vitro transcription of guide RNAs and 5'-triphosphate removal v2. (2023) doi:10.17504/protocols.io.n2bvjyp5vk5w/v17.
5. Zheng, G. X. Y. *et al.* Massively parallel digital transcriptional profiling of single cells. *Nat. Commun.* **8**, 14049 (2017).
6. Gu, W. *et al.* Depletion of Abundant Sequences by Hybridization (DASH): using Cas9 to remove unwanted high-abundance species in sequencing libraries and molecular counting applications. *Genome Biol.* **17**, 41 (2016).
